# Fair Funding Decisions: Consistency of the Time Horizons Used in the Calculation of Quality-Adjusted Life Years for Therapies for Very Rare Diseases by the National Institute for Health and Care Excellence in England

**DOI:** 10.3390/ijerph21050616

**Published:** 2024-05-13

**Authors:** Jasmin Barman-Aksözen, Nicole Hentschel, Mårten Pettersson, Eva Schupp, Francesca Granata, Cornelia Dechant, Mehmet Hakan Aksözen, Rocco Falchetto

**Affiliations:** 1 International Porphyria Patient Network (IPPN), Hegarstrasse 3, 8032 Zurich, Switzerland; 2 Independent Researcher, Hegarstrasse 3, 8032 Zurich, Switzerland; 3 Fondazione IRCCS Ca’ Granda Ospedale Maggiore Policlinico, S.C Medicina ad Indirizzo Metabolico, 20122 Milano, Italy

**Keywords:** health economics, quality-adjusted life years, National Institute for Health and Care Excellence, Highly Specialised Technologies programme, rare disease, erythropoietic protoporphyria

## Abstract

The National Institute for Health and Care Excellence (NICE) in England uses quality-adjusted life years (QALYs) to assess the cost-effectiveness of treatments. A QALY is a measure that combines the size of the clinical benefit of a treatment with the time the patient benefits from it, i.e., the time horizon. We wanted to know how consistently QALY gains are calculated at NICE. Therefore, we have analysed information on the time horizons used for the QALY calculations of the concluded evaluations conducted under the Highly Specialised Technologies programme for treatments of very rare diseases at NICE. For treatments with final guidance published by December 2023 (*n* = 29), a time horizon of median 97.5 years (range: 35 to 125 years) was used to calculate the QALY gains. For most QALY calculations, the accepted time horizon was longer than either the expected treatment duration or the estimated life expectancy. In contrast, for the only technology with a final negative funding decision, i.e., afamelanotide for treating the lifelong chronic disease erythropoietic protoporphyria, a time horizon that was shorter than the expected treatment duration was used. The fairness and consistency of the evaluation process of treatments for very rare diseases at NICE should be reviewed.

## 1. Introduction

**Figure 1:**
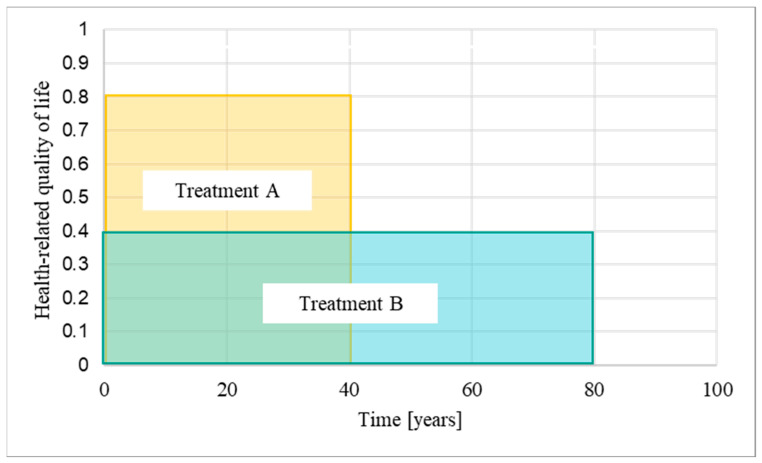
Quality-adjusted life years: A quality-adjusted life year (QALY) is a measure that combines the size of the clinical benefit of a treatment, measured as health-related quality of life, with the time over which the patient benefits from the treatment, i.e., the time horizon. The longer the time horizons, the more additional QALYs are accumulated [1] (modified). In the example provided in this figure, treatment A is associated with a quality of life of 0.8 (out of 1 maximum) assessed over a 40-year time horizon, while treatment B is associated with a quality of life of 0.4 and an 80-year time horizon. Treatment B therefore has the same QALY gain as treatment A.

A quality-adjusted life year (QALY) is a measure that combines the size of the clinical benefit of a treatment, measured as health-related quality of life, with the time over which the patient benefits from it, i.e., the time horizon [1] (Figure 1). The National Institute for Health and Care Excellence (NICE) in England use QALYs to assess the cost-effectiveness of treatments within and between diseases [2]. Treatments are considered cost-effective for use in the National Health Service (NHS) of England and Wales if they do not exceed a predefined threshold of around GBP 30,000 per QALY gained. Treatments with annual costs per patient that exceed this threshold are usually not recommended for funding.

**Figure 2:**
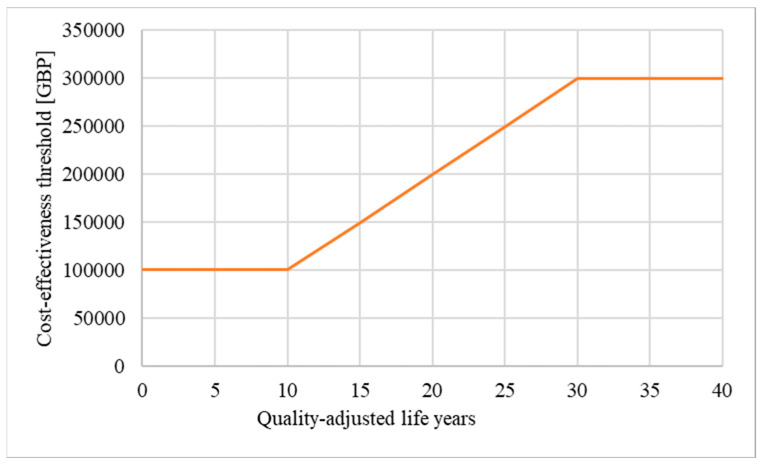
Weighting: If weighting is applied, the maximum price of a drug evaluated under the HST programme can increase from GBP 100,000 up to GBP 300,000 per QALY gained [13] (modified; own illustration).

In theory, a QALY is equal to a QALY, regardless of who benefits from an intervention [2,3]. However, critics have questioned the scientific validity of the concept and argue that limitations associated with the QALY methodology disadvantage certain patient populations, such as patients affected by chronic diseases, rare diseases, and the elderly [4,5,6]. To promote fairness in their decision making, NICE emphasises that the calculation of the QALY gain must be based on a methodologically sound, consistent, and transparent approach [2]. In addition, to ensure that societal preferences such as enabling access to innovative treatments for disadvantaged patient populations are considered, NICE regularly performs public consultations and adapts their methods [7]. For example, in 2013, NICE introduced the Highly Specialised Technologies (HST) programme for the evaluation of treatments for very rare diseases [8]. In Europe, a disease is defined as rare if it affects fewer than 1 person in 2000 [9]. The rarer a particular disease is, the more difficult it usually is to diagnose, manage, research, and develop treatments for [10,11]. To emphasise these additional challenges, diseases that affect fewer than 1 person in 50,000 are referred to as “ultra-rare” [9]. While NICE did not define “very rare” in their HST guideline, most technologies assessed under the HST programme are treatments for ultra-rare diseases [8]. Many drugs for rare diseases have higher-than-average prices and would not be considered cost-effective if assessed by NICE’s normal standards, and consequently, would not reach the patients [12]. As one of the measures, the HST programme allows a higher cost-effectiveness threshold. Moreover, the evaluating HST committee can consider a broader range of evidence than is permitted for more common diseases such as observational studies and qualitative data. In 2017, NICE defined the threshold for the cost-effectiveness of treatments assessed under the HST programme at GBP 100,000 per QALY gained. In addition, a QALY modifier, known as weighting, was implemented to reward treatments that provide significant benefits to patients (Table 1 and Figure 2): If a treatment assessed under the HST programme yields between 10 and 30 QALYs over the lifetime of the patients, the QALY gain can be weighted (i.e., multiplied) by a factor between 1 to 3. If applied, weighting increases the cost-effectiveness threshold from GBP 100,000 up to GBP 300,000 per QALY gained, allowing more expensive treatments to be funded by the NHS.

The time horizon is one of the factors that determines how many total QALYs are gained (Figure 1), and therefore influences whether weighting applies. We argue that it is important that the lengths of the time horizons are chosen in a consistent manner. The presented analysis focuses on the lengths of the time horizons accepted by the HST committee for all treatments evaluated between the start of the programme in 2013 and the end of December 2023, with special emphasis on the two evaluations in which our patient organisation, the International Porphyria Patient Network (IPPN), was involved, i.e., afamelanotide for treating erythropoietic protoporphyria (HST27) and givosiran for treating acute hepatic porphyria (HST16) [14,15].

## 2. Materials and Methods

### 2.1. Calculation of the QALY Gain and Determination of the Time Horizon

For this document analysis, we extracted information on the calculation of the QALY gains from publicly available documents at NICE.

#### 2.1.1. Calculation of the QALY Gain for the Evaluation at NICE 

For the calculation of the QALY gain for health economic analyses at NICE, data on the health-related quality of life of the patients need to be collected using the EQ-5D-3L questionnaire. Alternatively, other instruments with mapping algorithms to convert the obtained results into EQ-5D-3L data can be used [16]. In a next step, these health states must be transformed into corresponding utility values between 1 (best possible health) and 0 (dead) by using a national value set derived from studies in the general population to reflect societal preferences [17]. The obtained utility values are then multiplied with the time over which the patients benefit from the treatment, i.e., the time horizon. Because the data collected in clinical trials often do not cover all relevant aspects such as the entire treatment period, assumptions on, for example, the mean time of survival must be used to model the expected benefits and costs of treatments over time. In addition, in some circumstances, adjustments of the utility values, for example, for age or comorbidities may be needed. NICE further requires the discounting of the costs and benefits of treatment by 3.5% per year, or, in particular cases, 1.5% per year [2,18,19]. In their public documents on appraisal procedures, NICE in some cases reports the incremental, undiscounted QALY gain, while in other cases, the incremental, discounted QALY gains are provided.

#### 2.1.2. Determination of the Time Horizon at NICE

Over time, NICE has published several guidelines and additional supporting documents on how to calculate the QALY gain for their assessment. To understand how the time horizon should be determined, we extracted information from documents relevant to the assessment of treatments evaluated under the HST programme, i.e., “NICE health technology evaluations: the manual” [2], the “Interim Process and Methods of the Highly Specialised Technologies programme Updated to reflect 2017 changes” [8], the “Guide to the methods of technology appraisal 2013” [20], the “Guide to the processes of technology appraisal” [21] and the “Guide to the technology appraisal and highly specialised technologies appeal Process (2018)” [22]. All documents were downloaded from the NICE website in January 2020 and again in March 2024, to retrieve the most current versions. The initial calculation of the QALY gain of a treatment is usually prepared and submitted to NICE by the manufacturer of the drug [2,23]. The submission is then evaluated by an external evidence review group (sometimes called external assessment group), which can suggest alternative parameters for the calculation of the QALY gain. During the evaluation process, the feedback from the evidence review group, stakeholder groups and the public, e.g., individual patients and caregivers, is discussed at the committee meetings. Finally, the evaluating committee at NICE decides which parameters are accepted for the QALY calculation of the treatment under evaluation.

#### 2.1.3. Time Horizons Used in Concluded HST Appraisals at NICE

Between June 2020 and December 2023, we retrieved all documents for the concluded appraisal procedures of technologies evaluated under the HST programme from the “history” section of NICE’s website, i.e., the Final Evaluation Determination (FED) document, the committee papers, public committee slides of the committee meetings, the report of the evidence review group and, if applicable, the documents on the appeal processes. One researcher (JBA) extracted the information on the time horizons, whether weighting has been applied and which factor was used from the FED document. In case this information was not provided in the FED, the relevant sections in all other documents for a given appraisal were read to identify the parameters used for the final QALY calculation.

A second researcher (MHA) independently confirmed the findings. In case discrepancies were identified, they were discussed until an agreement was reached. All authors had access to the entire material, and critically assessed the analysis and its results and interpretation.

#### 2.1.4. Information on QALY Gains

In addition, for HST16 and HST27, information on the QALY gains was extracted from the appraisal documents. If not stated otherwise, QALY gains reported in this article are incremental, undiscounted QALY gains as published by NICE.

### 2.2. Statistical Analysis

Data with independent samples were analysed by unequal variances *t*-test using the *t*-test function in R [24]. A two-sided *p* ≤ 0.05 was considered statistically significant.

### 2.3. Data Availability

All material used for the presented analysis have been published by NICE.

## 3. Results

We first summarise information from the NICE guidelines on how the lengths of the time horizon used for the calculation of QALY gains should be determined (Section 3.1). Then, we present our findings on the lengths of the time horizons used to calculate the QALY gains of afamelanotide (Section 3.2) and givosiran (Section 3.3), together with background information on the disease characteristics and the evaluation process at NICE. In Section 3.4, we show the results of the comparison of all time horizons of treatments evaluated under the HST programme with final guidance published by end of December 2023.

### 3.1. Determination of the Time Horizon at NICE 

The “reference case”, which is part of the “Guide to the methods of technology appraisal”, contains the most detailed description on how to calculate QALY gains for the evaluation at NICE [2]. The reference case does not specify the length of the time horizon but states that it should be “long enough to reflect all important differences in costs or outcomes between the technologies being compared” (Table 2). The guidelines further clarify that the assumptions used for calculating the QALY gain need to be consistent between the technologies, should have external and internal validity, and must be transparently reported [2,20]. For treatments assessed under the HST programme, the “Interim Process and Methods of the Highly Specialised Technologies Programme Updated to reflect 2017 changes” applies in addition to the general guidelines [8]. According to this document, “the number of QALYs gained over the lifetime of patients” needs to be considered when assessing whether QALY weighting applies.

### 3.2. Afamelanotide: A Lifetime Time Horizon of 35 Years

Erythropoietic protoporphyria (EPP) is an inborn error of metabolism affecting the erythroid heme biosynthesis, leading to intolerance to visible light [25]. From their early childhood on, patients with EPP suffer from immediate and extremely painful phototoxic reactions in skin exposed to sunlight and certain artificial light sources, which can progress into second-degree burn injuries. Until 2014, no option to either treat or prevent these phototoxic reactions existed, and the management of the patients was limited to avoidance of exposure to visible light, which requires the patients adapt all aspects of daily living leading to low quality of life, social isolation, and mental health challenges [26,27]. In 2014, the European Medicines Agency (EMA) recommended approval of afamelanotide for use in adult patients as the first effective treatment to prevent phototoxic reactions in EPP [28,29]. Under treatment with afamelanotide, the patients have less frequent and less severe phototoxic reactions, an increased quality of life (as assessed with a disease-specific questionnaire) and a normalised time they spend in sunlight [30].

**Figure 3:**
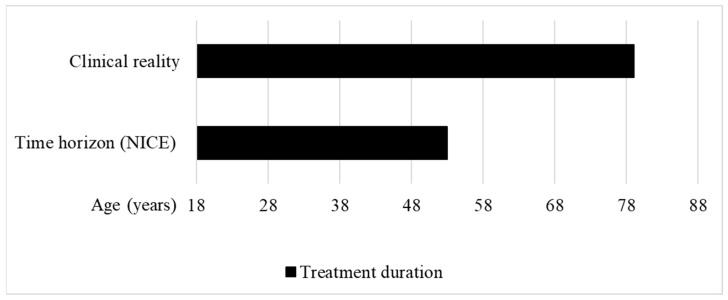
Treatment duration in the clinical reality vs. time horizon used to calculate the QALY gain of afamelanotide for treating erythropoietic protoporphyria at NICE. Clinical reality: The treatment duration in the clinical reality was set between the approved starting age of treatment at 18 years to the maximum published age of a patient treated with afamelanotide, i.e., 79 years [30]. Time horizon (NICE): The HST committee concluded that a time horizon of 35 years was reasonable to calculate the QALY gain of afamelanotide for treating EPP [14] (own illustration).

In 2016, NICE started to evaluate the cost-effectiveness of afamelanotide under the HST programme. The IPPN was involved in this evaluation as a stakeholder. In their initial assessment issued in May 2018, the HST committee concluded that, with 0.56 QALYs gained over the lifetime of the patients, the treatment would only provide small benefits, and did not recommend funding for afamelanotide [14]. For the calculation of the QALY gain, 35 years was assessed as a reasonable time horizon for treatment by the HST committee (Figure 3). However, except for rare hepatic complications, patients with EPP have a normal life expectancy.

Moreover, the 35-year time horizon does not reflect the clinical reality. The EMA recommends the use of afamelanotide in patients with EPP from age 18 to 70, with the additional requirement to monitor vital signs, and perform routine haematology and biochemistry, in patients older than 70 years of age [29]. In 2020, partial results of the ongoing real-world post-authorisation safety and efficacy study (PASS) conducted in treatment centres in the EU were published, showing that patients between 18.3 and 79.0 years of age are treated with afamelanotide (Figure 3) [30].

### 3.3. Givosiran: A 60-Year Time Horizon for a 13-Year Treatment Duration

The second evaluation at NICE, in which the IPPN was involved as stakeholder, concerned givosiran for the treatment of acute hepatic porphyria (AHP), a group of inborn errors of the heme biosynthesis in the liver [15,31]. Patients with AHP can experience extremely painful and debilitating acute attacks when exposed to triggering factors such as specific medications, hormones, stress, infections, and fasting, which may require hospitalisation and treatment with haem arginate, the standard of care [31]. However, for a subset of patients who experience frequent attacks despite avoiding triggering factors, the existing therapeutic options are not sufficient to effectively treat and prevent the symptoms [32]. In severe cases, a liver transplant can be performed, which eliminates the AHP but is associated with transplant-related health issues. Givosiran is a new treatment option for patients with AHP, and was shown to reduce the number and severity of acute attacks in patients with at least four acute attacks per year [33]. In 2020, givosiran was approved in the EU and subsequently evaluated by the HST committee [15].

**Figure 4:**
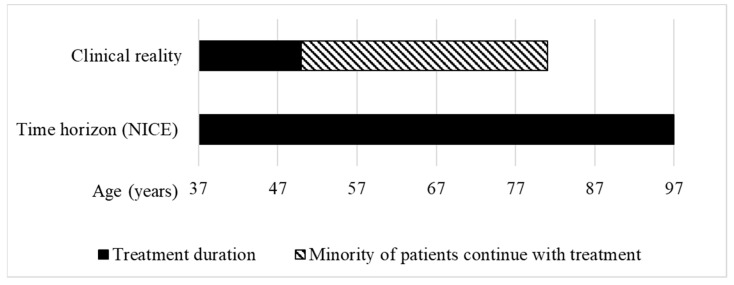
Treatment duration in the clinical reality vs. time horizon used to calculate the QALY gain of givosiran for treating acute hepatic porphyria at NICE. Clinical reality: According to the clinical experts, for most patients, starting age of treatment is 37 years and stopping age is 50 years [15]. Time horizon (NICE): The HST committee concluded that a time horizon of 60 years was reasonable to calculate the QALY gain, leading to a hypothetical life expectancy of the patients of 97 years (own illustration).

During the assessment of givosiran at NICE, “the clinical experts considered 37 years to be an accurate reflection of starting age” for treatment with givosiran, and that most people “stop treatment approximately by the age of 50” [15]. Only a minority of patients were assumed to continue with the treatment beyond age 50. For givosiran, the HST committee accepted an 18.6 QALY gain, calculated using a time horizon of 60 years (Figure 4). 

With a length of 60 years, the time horizon for givosiran is longer than the estimated treatment duration of 13 years for most patients, and would lead to a hypothetical life expectancy of up to 97 years. The World Bank estimates that the average life expectancy at birth of people living in the UK is 81 years [34] (Figure 4). With over 10 QALYs gained, givosiran was assessed by the HST committee as eligible for QALY weighting with a factor of 1.8 and recommended for funding in the NHS.

### 3.4. Time Horizons of Treatments Evaluated under the HST Programme

In the IPPN’s understanding, either the time horizon for the QALY calculation of afamelanotide was unreasonably short, or the time horizon in the case of givosiran was disproportionally long. To better understand their standard approach, we analysed the time horizons accepted in previous evaluations conducted by the HST committee.

#### 3.4.1. Time Horizons

We identified 29 evaluations with final guidance published via the NICE website by 31 December 2023, concerning 26 different treatments (Table 3) (the evaluations HST2, HST3 and HST6 have been reviewed and replaced with the updated guidance HST19, HST22 and HST23, respectively). Form these 29 evaluations, information on the time horizon could be identified for 24 (83%), whereas for 5 cases, this information was not provided, but only described as a “lifetime time horizon” because the length of the time horizon was, for example, deemed confidential for commercial reasons.

The median time horizon used to calculate the QALY gain of treatments for which information on the length of the time horizon was provided (*n* = 24) was 97.5 years, with a range of 35 to 125 years. The mean time horizon was 79.6 (SD = 26.7). In 12 of the evaluations, time horizons ≥ 100 years have been used to calculate the QALY gains. However, even if time horizons below the average length were used to calculate the QALY gains, the assumed treatment durations did not always appear realistic. For example, for eliglustat for treating type 1 Gaucher disease (HST5), a time horizon of 70 years was used to calculate the QALY gain, despite an expected age for starting with the treatment of between 32 to 38 years [35]. Interestingly, all QALY gains for gene therapies with publicly available information (*n* = 4) were calculated using a time horizon of 100 years. In our assessment, in at least 15 (63%) of the 24 evaluations, time horizons exceeding either the expected treatment durations or the estimated life expectancy of 81 years were accepted by the HST committee.

#### 3.4.2. Association between Time Horizon and Application of the QALY Modifier Weighting

The HST programme started in 2013, but weighting was only introduced in 2017, after the evaluation of the first six technologies had been concluded (Table 3). Of the 23 treatments that were evaluated after the introduction of weighting, a total of 12 treatments were assessed as eligible for weighting, whereby the factor was not specified in seven cases.

**Figure 5:**
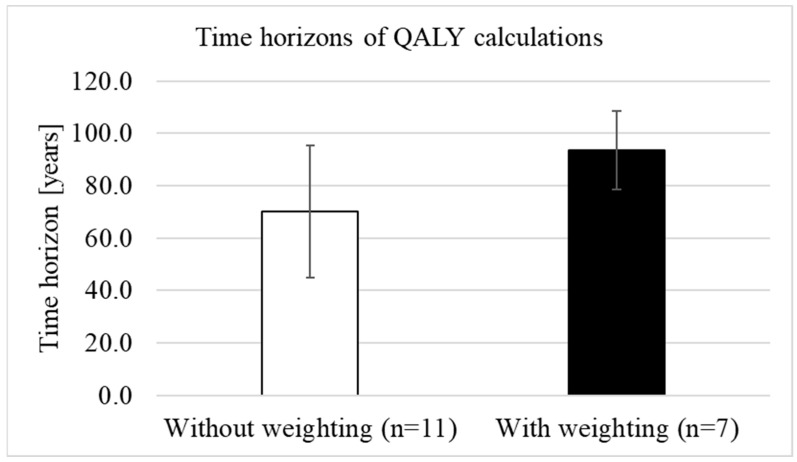
Difference in the lengths of the time horizons between treatments with and without weighting (own illustration).

We analysed whether QALY weighting was associated with longer time horizons. The mean time horizon before the introduction of weighting (*n* = 6) was 80.8 years (SD = 35.4) with a median of 85 years (range: 35 to 125 years) and was slightly, but not statistically significantly (*p* = 0.920) longer than the mean time horizon after the introduction of weighting (*n* = 18), which was (mean) 79.2 years (SD = 24.3) with a median of 97.5 years (range: 40 to 100). When only comparing the time horizons after the introduction of weighting, the mean length without weighting (*n* = 11) was 70 years (SD = 25.2; median 60 years, range: 40–100), while for treatments with weighting (*n* = 7), the mean time horizon was 93.6 years (SD = 15; median 100 years, range: 60–100) (Figure 5). The differences in the lengths of the time horizons between treatments with and without weighting were statistically significant (*p* = 0.024).

## 4. Discussion

Our analysis shows that with a median of 97.5 years, most time horizons accepted for calculating the QALY gains of treatments evaluated under the HST programme are longer than either the expected treatment duration or the estimated general life expectancy of 81 years (Section 3.4.1). Longer time horizons lead to higher QALY gains (Figure 1) and, in our assessment, can impact the fairness of funding decisions in two ways [36]:

Firstly, unreasonably long time horizons lead to higher total QALY gains, creating the impression that these treatments are more beneficial than treatments with realistic time horizons (and QALY gains). According to the reference case, the length of the time horizon should “reflect all important differences in costs or outcomes between the technologies being compared” (Section 3.1). NICE compares treatments within and across different disease areas. However, if the lengths of the time horizons of treatments are inconsistently determined, the resulting QALY gains do not objectively reflect the benefits of the treatments and a direct comparison is not possible. 

Secondly, in the case of treatments assessed under the HST programme, longer time horizons can result in higher drug prices. If a treatment provides more than 10 QALYs, the QALY modifier weighting can be applied, which increases the cost-effectiveness threshold from GBP 100,000 up to GBP 300,000 per QALY gained (Figure 2). Because of weighting, unreasonably long time horizons can lead to higher drug prices, which divert resources from other areas of the healthcare system. Our analysis indeed demonstrates that treatments with longer time horizons were more likely assessed by the HST committee to be eligible for weighting than treatments with shorter time horizons (Section 3.4.2).

According to the “Interim Process and Methods of the Highly Specialised Technologies Programme” (Section 3.1), a lifetime time horizon should be used to calculate the QALY gain [8]. However, for the only technology with a final negative funding decision, i.e., afamelanotide for treating EPP, a time horizon of 35 years, which is considerably shorter than the expected treatment duration or life expectancy of the patients, was assumed as a reasonable treatment duration by the HST committee (Section 3.2). In 2022, we presented a preliminary analysis of the time horizons in evaluations conducted under the HST programme to the committee, who later adapted the time horizon for the calculation of the QALY gain of afamelanotide to 60 years [37]. While more realistic, a 60-year time horizon does not cover the entire expected treatment duration with afamelanotide up to age 81 years, and is still considerably shorter than the median 97.5-year time horizon accepted in the previous evaluations. In March 2023, the HST committee assessed that with a QALY gain of just below 10, afamelanotide was not cost-effective for use in the NHS [14]. Until now, patients suffering from EPP living in England and Wales do not have access to the only treatment for their condition.

Patients affected by rare diseases already face considerable challenges in receiving adequate healthcare services, with long delays in the time to diagnosis, limited knowledge about the pathophysiology and natural history of their conditions, a lack of healthcare specialists and research interests, and, for about 95% of the diseases, no specific treatment option available [9,10,11]. Even when approved by regulatory bodies, before becoming accessible to patients, treatments have to be re-evaluated by national health technology assessment authorities such as NICE, which usually have different criteria and thresholds to determine the cost-effectiveness of drugs—criteria that have been developed for more common diseases, where evidence generation is typically more straightforward [11,12]. Adjustments of the assessment process, such as the HST programme in England, are therefore necessary and important to promote fair access to treatment as well as prevent unreasonable negative funding decisions, which can have devastating consequences for patients without alternative treatment options. Our data question the fairness and consistency of the evaluation process of treatments for very rare diseases at NICE. Beyond the impact on particular patient groups, we believe that accepting unreasonably short or long time horizons of up to 125 years for QALY calculations without providing a scientifically sound rationale can undermine trust in NICE as an institution [38]. Moreover, in our assessment, in-depth analyses of additional parameters and assumptions for the QALY calculations and other aspects of the evaluation process might reveal further evidence of inconsistencies between the appraisals conducted by the HST committee. For example, during our analysis, we noticed differences in whether patient and medical expert testimonies were included in decision-making.

The limitations of our study are the relatively small sample size of only 29 technologies, and changes in the procedure after the start of the HST programme, i.e., the introduction of weighting in 2017. In addition, the lack of access to the full information on how the QALY gain has been calculated, i.e., the economic models, prevents a comprehensive analysis and comparison of the QALY gains. Moreover, our study has revealed that for 7 (24%) of the 29 evaluations, even basic information such as the lengths of the time horizon or the factor that was applied for weighting was not provided. Another limitation of our study is that we did not interview members of the HST committee to better represent their views. However, since the International Porphyria Patient Network is a stakeholder in two of the appraisal proceedings, we have already discussed several of the presented aspects at committee meetings or in writing, and have summarised the reactions of the committee in our article (Section 3.2 and Section 3.3).

A strength of our study is that the time horizon can be considered a relatively unambiguous and easily accessible parameter of the QALY calculation. It is, for example, clear that a time horizon of 35 years in a disease that is not life-limiting does not allow for counting benefits over the full lifetime of the patients, and therefore underestimates the total QALY gain. Another strength of the study is that it only concerns decisions taken by one committee at NICE, which in addition was chaired by the same person for the entire study period. Because of this continuity, a high degree of consistency between the appraisals should have been expected.

## 5. Conclusions

Our analysis suggests that the time horizons used for the calculation of the QALY gains of treatments assessed under NICE’s HST programme are not consistently determined. Since the total QALY gain directly influences whether a treatment is considered as cost effective for use in the NHS and should be provided to the patients living in England and Wales, fair funding decisions are currently not guaranteed. Accepting time horizons that are longer than the treatment duration or life expectancy of the patients, i.e., up to 125 years, appears to contrast with NICE’s guidelines, which require a consistent and methodologically sound approach to evaluating drugs.

Our analysis is under no circumstances meant to compromise access to treatments for patients with very rare diseases with an already positive recommendation for funding by NICE. However, to ensure fairness and trust in their decision-making, NICE should consider reviewing the decisions taken by the HST committee, explain the reasons for the differences in time horizons, and correct parameters for QALY calculations that are based on demonstrably irrational conclusions. In addition, we believe that NICE should also review whether declaring parameters for the QALY calculation such as the length of the time horizon as confidential information is compatible with their own policy to transparently report QALY gains.

Finally, we hope that our work will prompt further investigations by other researchers, and stimulate the public debate on fair treatment within the healthcare system.

## Figures and Tables

**Table 1:** Incremental QALYs gained and associated weight. For treatments assessed under the Highly Specialised Technologies (HST) programme for very rare diseases, a QALY modifier called weighting can be applied for treatments that have gained between 10 and 30 QALYs over the patients’ lifetimes [8] (p. 12, modified; own compilation).

Incremental QALYs Gained (per Patient, Using Lifetime Horizon)	Weight
Less than or equal to 10	1
11–29	Between 1 and 3 (using equal increments)
Greater than or equal to 30	3

**Table 2:** Instructions on how to determine the time horizon for NICE appraisals (own compilation).

Guideline	Instruction
NICE health technology evaluations: the manual. Reference case	“Time horizon: Long enough to reflect all important differences in costs or outcomes between the technologies being compared” [2] (p. 67).
NICE health technology evaluations: the manual	“Modelling is often needed to extrapolate costs and health benefits over an extended time horizon. Assumptions used to extrapolate the treatment effect over the relevant time horizon should have both external and internal validity and be reported transparently” [2] (p. 87).
Guide to the methods of technology appraisal 2013	“The Institute has to make decisions across different technologies and disease areas. It is, therefore, crucial that analyses of clinical and cost effectiveness undertaken to inform the appraisal adopt a consistent approach” [20] (p. 34).
Interim Process and Methods of the Highly Specialised Technologies Programme Updated to reflect 2017 changes	“Depending on the number of QALYs gained over the lifetime of patients, when comparing the new technology with its relevant comparator(s), the committee will apply a weight between 1 and 3, using equal increments, for a range between 10 and 30 QALYs gained” [8] (p. 12).

**Table 3:** Evaluations of treatments under the Highly Specialised Technologies (HST) programme at NICE with final guidance published between the start of the programme in 2013 and December 2023 (own compilation).

Assessment	Technology	Time Horizon [Years]	QALY Modifier (Weighting)
HST1	Eculizumab for the treatment of atypical haemolytic uraemic syndrome (aHUS)	125	Before introduction
HST2 (=HST19 ^R^)	Elosulfase alfa for treating mucopolysaccharidosis type IVA	100	Before introduction
HST3 (=HST22 ^R^)	Ataluren for treating Duchenne muscular dystrophy with a nonsense mutation in the dystrophin gene	35	Before introduction
HST4	Migalastat for treating Fabry disease	48	Before introduction
HST5	Eliglustat for treating type 1 Gaucher disease	70	Before introduction
HST6 (=HST23 ^R^)	Asfotase alfa for treating paediatric-onset hypophosphatasia	106.7	Before introduction
HST7 ^G^	Strimvelis for treating severe combined immunodeficiency caused by adenosine deaminase deficiency	100	Applied, factor 1.4
HST8	Burosumab for treating X-linked hypophosphataemia	Lifetime time horizon	Applied, factor not given
HST9	Inotersen for treating hereditary transthyretin amyloidosis	41	Not applied
HST10	Patisiran for treating hereditary transthyretin amyloidosis	40	Not applied
HST11 ^G^	Voretigene neparvovec for treating inherited retinal dystrophies caused by RPE65 gene mutations	100	Applied, factor 1.2
HST12	Cerliponase alfa for treating neuronal ceroid lipofuscinosis type 2	95	Applied, factor 3
HST13	Volanesorsen for treating familial chylomicronaemia syndrome	59	Not applied
HST14	Metreleptin for treating lipodystrophy	100	Not applied
HST15 ^G^	Onasemnogene abeparvovec for treating type 1 spinal muscular atrophy	Lifetime time horizon	Applied, factor not given
**HST16**	**Givosiran for treating acute hepatic porphyria**	**60**	**Applied, factor 1.8**
HST17	Odevixibat for treating progressive familial intrahepatic cholestasis	100	Not applied
HST18 ^G^	Atidarsagene autotemcel for treating metachromatic leukodystrophy	100	Applied, factor between 1 and 3
HST19 ^R^ (=HST2)	Elosulfase alfa for treating mucopolysaccharidosis type 4A (review of HST2)	100	Applied, factor not given
HST20	Selumetinib for treating symptomatic and inoperable plexiform neurofibromas associated with type 1 neurofibromatosis in children aged 3 and over	100	Not applied
HST21	Setmelanotide for treating obesity caused by LEPR or POMC deficiency	100	Applied, factor not given
HST22 ^R^ (=HST3)	Ataluren for treating Duchenne muscular dystrophy with a nonsense mutation in the dystrophin gene (review)	70	Not applied
HST23 ^R^ (=HST6)	Asfotase alfa for treating paediatric-onset hypophosphatasia	Lifetime time horizon	Applied, factor not given
HST24 ^G^	Onasemnogene abeparvovec for treating presymptomatic spinal muscular atrophy	100	Not applied
HST25	Lumasiran for treating primary hyperoxaluria type 1	Lifetime time horizon	Applied, factor 2.0
HST26 ^G^	Eladocagene exuparvovec for treating aromatic L-amino acid decarboxylase deficiency	Lifetime time horizon	Applied, factor confidential
**[HST27]**	**Afamelanotide for treating erythropoietic protoporphyria**	**35 (2018)** **60 (2023)**	**Not applied**
HST28	Birch bark extract for treating epidermolysis bullosa	50	Not applied
HST29	Velmanase alfa for treating alpha-mannosidosis	50	Not applied

In bold: Evaluations in which the IPPN was a stakeholder: Treatments not recommended for funding. Before introduction: Treatments that were evaluated before the introduction of weighting in 2017. ^R^: Review of the original evaluation. ^G^: Gene therapy.

## Data Availability

All material and information used for the analysis have been published by NICE and are available from the website. Documents that meanwhile have been removed from the website can be accessed via a Freedom of Information Act request directed towards NICE.

## Researcher Characteristics

The International Porphyria Patient Network (IPPN) is a not-for-profit organisation of patients and carers with a professional background in science, medicine, economics, statistics, and other relevant expertise. The IPPN provides support and counselling for patients suffering from one of the eight forms of porphyria, and collaborates with national and international porphyria associations and individual patients in scientific, medical, and healthcare policy matters. To date, we have funded our activities exclusively out-of-pocket, minimising any potential dependence on third parties.
